# Metagenomic Annotation Networks: Construction and Applications

**DOI:** 10.1371/journal.pone.0041283

**Published:** 2012-08-07

**Authors:** Gregory Vey, Gabriel Moreno-Hagelsieb

**Affiliations:** 1 Department of Biology, University of Waterloo, Waterloo, Ontario, Canada; 2 Department of Biology, Wilfrid Laurier University, Waterloo, Ontario, Canada; Swedish University of Agricultural Sciences, Sweden

## Abstract

The derivation and comparison of biological interaction networks are vital for understanding the functional capacity and hierarchical organization of integrated microbial communities. In the current work we present metagenomic annotation networks as a novel taxonomy-free approach for understanding the functional architecture of metagenomes. Specifically, metagenomic operon predictions are exploited to derive functional interactions that are translated and categorized according to their associated functional annotations. The result is a collection of discrete networks of weighted annotation linkages. These networks are subsequently examined for the occurrence of annotation modules that portray the functional and organizational characteristics of various microbial communities. A variety of network perspectives and annotation categories are applied to recover a diverse range of modules with different degrees of annotative cohesiveness. Applications to biocatalyst discovery and human health issues are discussed, as well as the limitations of the current implementation.

## Introduction

The ubiquity of next-generation sequencing projects has vastly accelerated the accumulation of metagenomic sequence data. As of last year the Sequence Read Archive [1] exceeded 100 Terabases of open-access reads produced by next-generation sequencing efforts [2]. A common goal in attempting to understand the functional capabilities of newly sequenced microbial communities involves the annotation of putative genes through the assignment of biological functions. Such functional annotation relies heavily on homology-based annotation transfer using tools such as BLAST, HMMs, and motif finding algorithms [3]. In turn, the success of these approaches is necessarily bounded by the diversity of the reference databases that are used to find candidate annotations. However, an important proportion of microorganisms do not grow under common laboratory culturing conditions [4], [5]. This limited spectrum of cultured microbial diversity, combined with biases in applied research interests, has yielded a skewed representation within sequence annotation databases [6]. Because metagenomes represent an attempt to gain access to the uncultured majority, homology-based annotation methods rooted in limited experimental knowledge about the functional roles of gene products are insufficient to adequately address the influx of unknown genes [7].

Given the difficulties in the annotation of individual metagenomic genes, the derivation and comparison of biological interaction networks represents a promising prospect for metagenomic data sources. Nevertheless, interaction networks can reveal vital information about functional organization and activity [8]. For example, studies of interaction networks in *Escherichia coli* [9] and *Saccharomyces cerevisiae* [10] have provided a systems perspective of these genomes by enumerating their respective functional modules. Recently there have been several attempts to capture metagenomic analogs of traditional interaction networks through the prediction of metabolic pathways and functional modules. MetaPath [11] uses prior knowledge of metabolic pathways in conjunction with metagenomic sequence data to predict the occurrence of metabolic pathways in metagenomic data sources. In contrast, Konietzny *et al.* [12] used a Bayesian approach to find co-occurrence patterns for functional descriptors contained in microbial genome annotations in order to infer functional modules.

**Figure 1:**
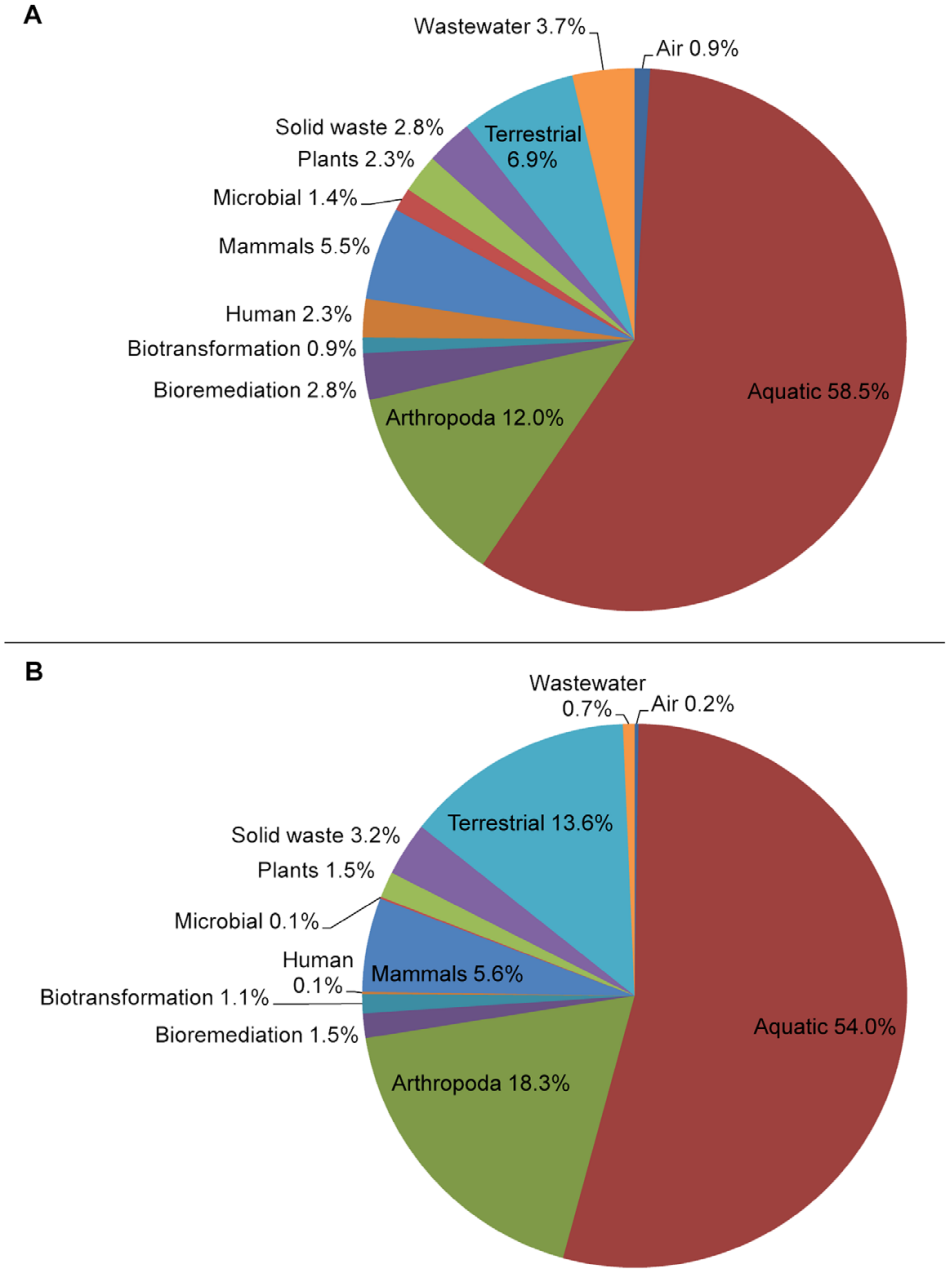
Data Source Diversity. The relative proportions (%) of various data source types that were used are shown categorized according to IMG/M microbiome taxa at the class level. Panel A shows the proportions (%) with respect to the total number of data sets while Panel B shows the proportions (%) with respect to the total number of genes.

In the present work we exploit metagenomic operon predictions to derive functional interactions that are translated and categorized according to their associated functional annotations as provided in the IMG/M metagenomics database [13] (Figure 1). The result is a collection of discrete networks of weighted annotation linkages that are subsequently examined for the occurrence of annotation modules that portray the functional and hierarchical organization of various microbial communities. A variety of network perspectives and annotation categories are compared and their potential applications are discussed.

## Results

**Figure 2:**
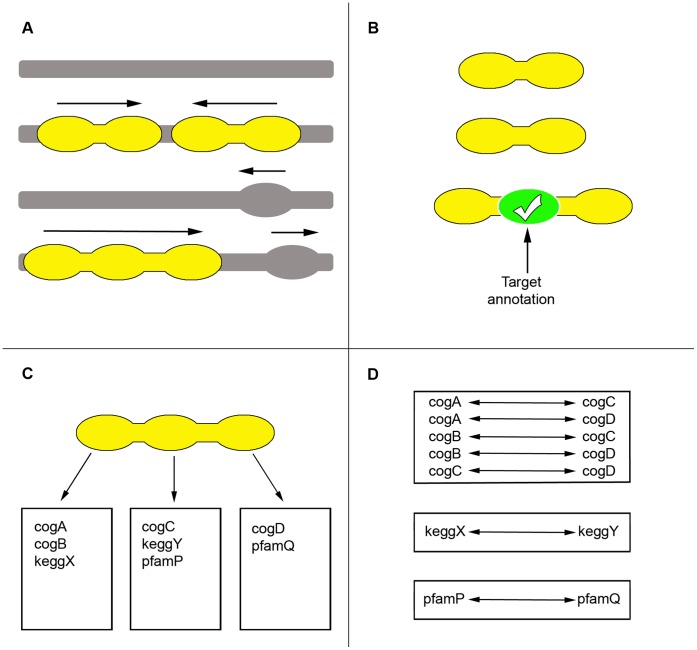
Network Construction Workflow. Operonic genes are predicted on the basis of co-direction and intergenic proximity using scaffolds containing more than one gene (Panel A). Operons and their constituent genes can be filtered according to the presence or absence of a target annotation such that at least one member of an operon is required to possess a target descriptor (Panel B). Note that the filter step is optional and can applied to obtain target perspective networks while being omitted in the construction of source perspective networks. Each gene in a given operon is mined for its various types of functional annotations where any particular type has a domain of existing values (Panel C). For each operon, the obtained functional annotations are used to infer bidirectional functional interactions for annotations having the same type but different values (Panel D). Note that interactions are inferred directly for immediately adjacent gene pairs and also transitively for downstream members within the same operon.

In order to demonstrate the utility of metagenomic annotation networks, networks employing a variety of perspectives and annotation categories were constructed and compared. The network construction protocol proposed in this work is confined to a process of network prediction followed by network translation (Figure 2; see also Methods). Subsequent analyses of the resulting networks were performed in order to demonstrate potential uses and applications but not as part of the network construction protocol itself. Therefore, examples provided here involve the use of Cytoscape 2.8.1 [23] for network analyses and the MINE plugin [24] for the identification of putative annotation modules. However, these tools were selected on the basis of potential familiarity for readers and it is certainly possible to use any other software, plugins, or algorithms as required for particular investigations.

**Figure 3:**
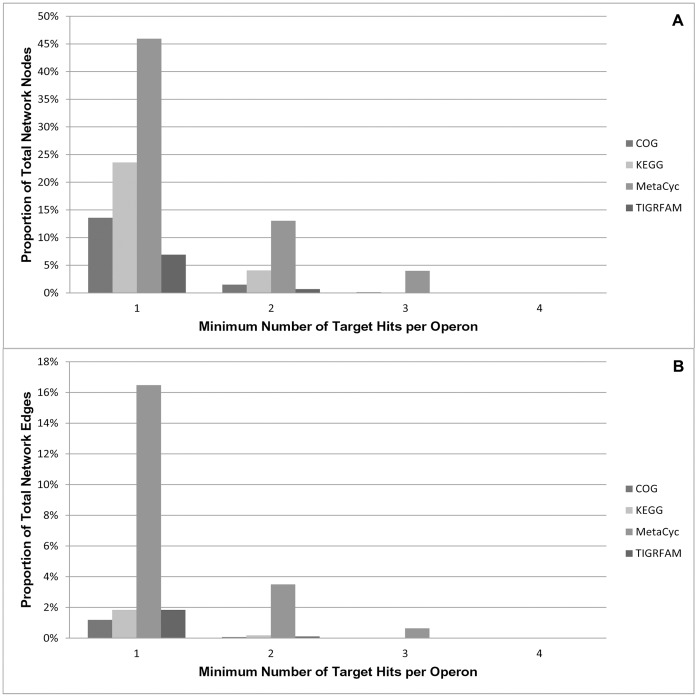
Target Stringency versus Network Coverage. Four polyketide target perspective networks were constructed with progressively increasing target stringency and each network was translated into each of the four annotation categories. The proportion of nodes and edges in each polyketide network was compared to its corresponding overall network. Panel A shows that coverage for nodes decreased for all annotation categories with increasing target stringency and Panel B shows that coverage for edges also decreased for all annotation categories with increasing target stringency.

### Target Perspective Networks

Networks can be constructed from a target perspective by using a keyword or series of keywords joined by logical operators to filter and reduce a set of results on the basis of keyword occurrence, or target hits. The goal is to constrain the resulting functional interactions so that they reflect a target-centric view for a domain of interest, such as interactions relating to cellulases. In the following examples single keywords of general interest, namely “polyketide” and “cellulase,” were used to select specific operons from the complete set of available operons thereby reducing the overall network into specific target perspective networks. However, it is possible to construct a target perspective network from a smaller and more specific range of data sets, such as using only human gut microbiomes (see Source Perspective Networks).

**Table 1:** Summary of Network Features.

Network Type	Annotation	Perspective	Nodes	Edges	Modules
Cellulase	MetaCyc	Target	213	779	5
Cellulase	COG	Target	301	763	33
Human Gut	KEGG	Source	153	192	11
Human Gut	TIGRFAM	Source	543	607	57
Gut Intersection	TIGRFAM	Comparative	407	278	19
Gut Difference	TIGRFAM	Comparative	356	329	20

The general features of each metagenomic functional network are shown including the type of network, the category of annotations used to construct the network, the network perspective, the number of nodes and edges that compose the network, and the number of predicted functional modules contained within the network.

Prior to evaluating any target perspectives networks, the effects of target stringency were investigated. Specifically, operons can be qualified as target hits if a fixed number or scalable proportion of their member genes have an annotation that contains the target. To determine the effects of target stringency versus network coverage, four polyketide target perspective networks were constructed and the stringency for qualification was progressively increased. Operons in the first network were required to have at least one target hit, operons in the second network were required to have at least two target hits, and so on, up to and including a stringency of requiring at least four target hits. Furthermore, each of the four networks was translated into each the four different annotation categories (see Methods) resulting in four sets of four target perspective networks. Figure 3 shows the proportion of nodes and edges recovered from the equivalent overall network (i.e. no filtering) with respect to increasing target stringency across each annotation category. The results illustrated that coverage for both nodes and edges decreased for all annotation categories with increasing target stringency. Therefore, the target perspective networks that follow used the least stringent requirement (i.e. at least one target hit) in an attempt to maximize the diversity and number of putative functional interactions available for subsequent analyses.

**Figure 4:**
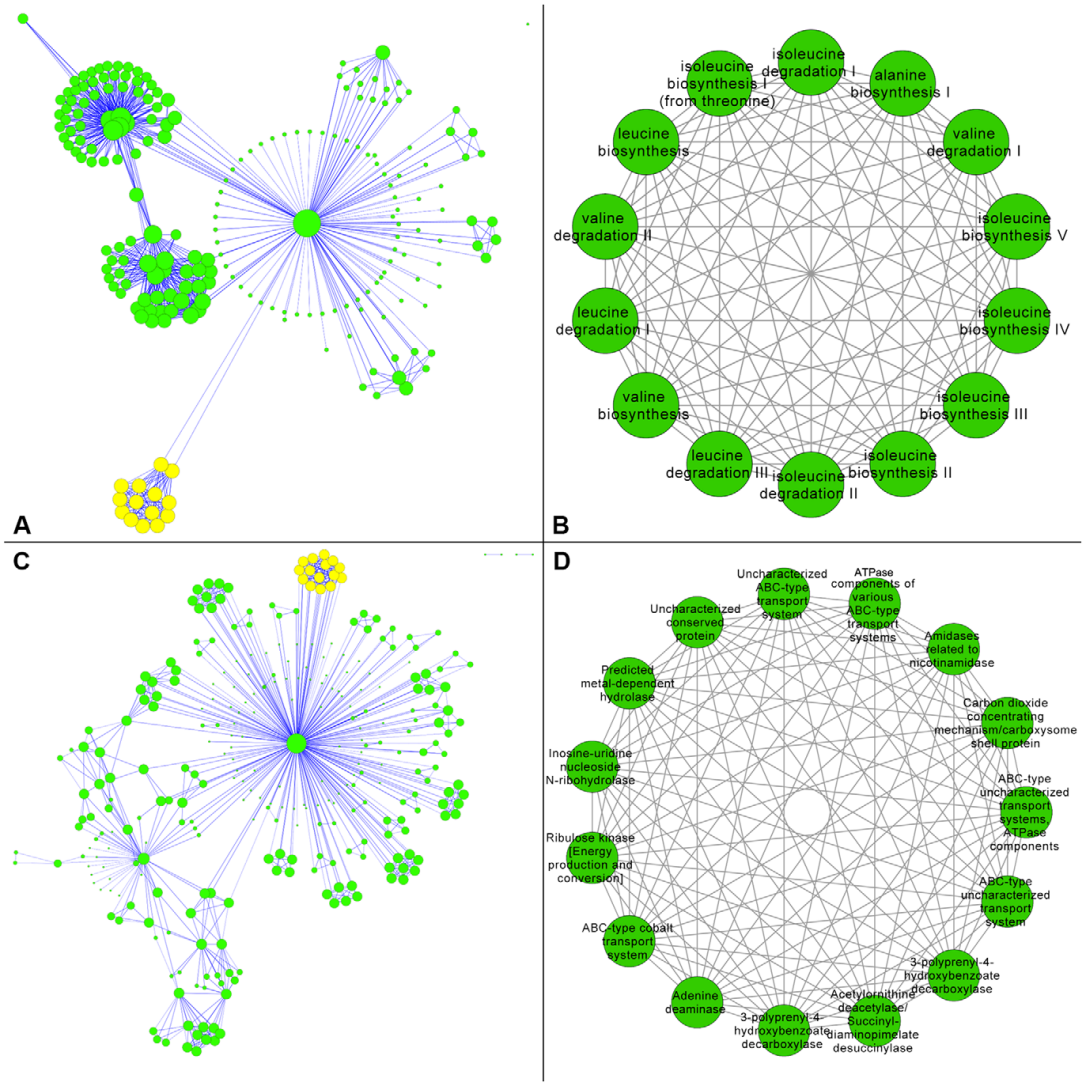
Metagenomic Cellulase Networks. The target perspective networks for cellulase functional interactions are shown where large node diameter represents high node degree within each respective network. Panel A shows a network constructed using MetaCyc annotations with a highly connected central hub having the annotation PWY-1001: cellulose biosynthesis. The highlighted nodes represent the top ranking module which is enlarged in Panel B. Panel C shows a network constructed using COG annotations and features a highly connected central hub with the annotation COG1363: cellulase M and related proteins. The highlighted nodes represent the top ranking module which is enlarged in Panel D.

**Figure 5:**
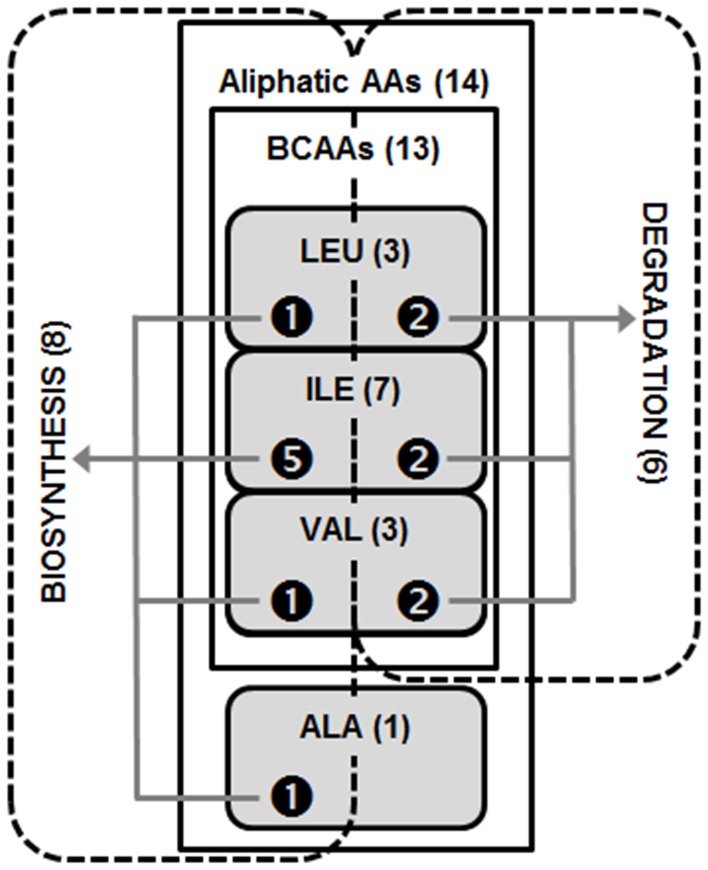
Thematic Set Diagram. The annotative themes for the top ranked MetaCyc module are depicted where the numeric values indicate the number of annotations belonging to a thematic category. Specifically, amino acid categories are represented vertically and metabolic categories are represented horizontally. Note, the vertical themes are encapsulatory while the horizontal themes are mutually exclusive. A variety of functional perspectives can be simultaneously visualized by way of the interacting and overlapping thematic sets.

A MetaCyc cellulase network was constructed that consisted of 213 nodes and 779 edges (Table 1). A highly connected central hub was observed that had the annotation PWY-1001: cellulose biosynthesis (Figure 4, Panel A). Five modules were identified within the network (Tables S1 and S2). The top ranked module (Figure 4, Panel B) contained annotations relating to amino acid degradation and biosynthesis (Table 2). The precise annotation terms were analyzed for more general themes and a highly cohesive module emerged that described aliphatic amino acid metabolism, with particular emphasis on branched-chain amino acids (BCAAs) (Figure 5). BCAA metabolism is consistent with functional expectations for ruminal bacteria such as members of the genus *Peptostreptococcus* [14]. Likewise, data from ruminal environments would be expected to contribute interactions to a metagenomic cellulase network.

**Table 2:** Top Ranked Functional Modules.

Source	Score	Nodes	Edges	Members
MetaCyc Cellulase Network	14.0	14	91	Alanine biosynthesis I; Isoleucine biosynthesis I (from threonine); Isoleucine biosynthesis II; Isoleucine biosynthesis III; Isoleucine biosynthesis IV; Isoleucine biosynthesis V; Isoleucine degradation I; Isoleucine degradation II; Leucine biosynthesis; Leucine degradation I; Leucine degradation III; Valine biosynthesis; Valine degradation I; Valine degradation II
COG Cellulase Network	15.0	15	105	3-polyprenyl-4-hydroxybenzoate decarboxylase; 3-polyprenyl-4-hydroxybenzoate decarboxylase and related decarboxylases; ABC-type cobalt transport system, permease component CbiQ and related transporters; ABC-type uncharacterized transport system, permease component; ABC-type uncharacterized transport systems, ATPase components; ATPase components of various ABC-type transport systems, contain duplicated ATPase; Acetylornithine deacetylase/Succinyl-diaminopimelate desuccinylase and related deacylases; Adenine deaminase; Amidases related to nicotinamidase; Carbon dioxide concentrating mechanism/carboxysome shell protein; Inosine-uridine nucleoside N-ribohydrolase; Predicted metal-dependent hydrolase; Ribulose kinase; Uncharacterized ABC-type transport system, permease component; Uncharacterized conserved protein
KEGG Human GutNetwork	6.2	13	37	Bacitracin transport system; C5 isoprenoid biosynthesis, mevalonate pathway; Ceramide biosynthesis; Eicosanoid biosynthesis, arachidonate = >8(S)-HETE; Gluconeogenesis, oxaloacetate = > fructose-6P; Glycolysis (Embden-Meyerhof pathway), glucose = >pyruvate; Glycolysis, core module involving three-carbon compounds; Indolepyruvate:ferredoxin oxidoreductase; Non-phosphorylative Entner-Doudoroff pathway, gluconate = > glyceraldehyde + pyruvate; Phosphatidylcholine (PC) biosynthesis, PE = >PC; Phosphatidylethanolamine (PE) biosynthesis, ethanolamine = >PE; Pyruvate oxidation, pyruvate = > acetyl-CoA; Semi-phosphorylative Entner-Doudoroff pathway, gluconate = > glyceraldehyde-3P + pyruvate
TIGRFAM Human Gut Network	8.2	18	70	30S ribosomal protein S17; 50S ribosomal protein L3, bacterial; 50S ribosomal protein L4, bacterial/organelle; Preprotein translocase, SecY subunit; Ribosomal protein L14, bacterial/organelle; Ribosomal protein L15, bacterial/organelle; Ribosomal protein L16, bacterial/organelle; Ribosomal protein L18, bacterial type; Ribosomal protein L2, bacterial/organellar; Ribosomal protein L22, bacterial type; Ribosomal protein L24, bacterial/organelle; Ribosomal protein L29; Ribosomal protein L30, bacterial/organelle; Ribosomal protein L6, bacterial type; Ribosomal protein S10, bacterial/organelle; Ribosomal protein S19, bacterial/organelle; Ribosomal protein S3, bacterial type; Ribosomal protein S5, bacterial/organelle type
Human Gut IntersectionNetwork	8.2	13	49	30S ribosomal protein S17; 50S ribosomal protein L3, bacterial; 50S ribosomal protein L4, bacterial/organelle; Ribosomal protein L14, bacterial/organelle; Ribosomal protein L16, bacterial/organelle; Ribosomal protein L18, bacterial type; Ribosomal protein L2, bacterial/organellar; Ribosomal protein L22, bacterial type; Ribosomal protein L24, bacterial/organelle; Ribosomal protein L29; Ribosomal protein L6, bacterial type; Ribosomal protein S19, bacterial/organelle; Ribosomal protein S3, bacterial type
Human Gut DifferenceNetwork	7.7	8	27	50S ribosomal protein L14P; 50S ribosomal protein L30P, archaeal; Archaeal ribosomal protein S17P; Ribosomal protein L24p/L26e, archaeal/eukaryotic; Ribosomal protein L29; Ribosomal protein S3, eukaryotic/archaeal type; Ribosomal protein S5(archaeal type)/S2(eukaryote cytosolic type); Translation initation factor SUI1, putative, prokaryotic

The general features of the highest scoring functional module from each network are shown including the source network, the score assigned by MINE, the number of nodes and edges that compose the module, and the member annotations derived from the nodes. Member annotations are sorted in ascending lexicographical order and delimited using the semicolon symbol. For all top ranked modules each member is unique.

A COG cellulase network was constructed that consisted of 301 nodes and 763 edges (Table 1). A highly connected central hub was observed that had the annotation COG1363: cellulase M and related proteins (Figure 4, Panel C). A total of 33 modules were identified within the network (Tables S1 and S2). The top ranked module (Figure 4, Panel D) contained annotations relating to ABC-type transport, permease, ATPase, as well as various other terms (Table 2). Furthermore, the term “uncharacterized” was observed in conjunction with several instances of the previously listed annotations. The precise annotation terms were analyzed for more general themes resulting in a less cohesive module than the top ranked MetaCyc module. Nevertheless, these annotations are generally consistent with secretion and transfer activities such as multienzyme secretion in the cellulolytic bacterium *Clostridium thermocellum* [15] and glycoside hydrolase secretion in *Thermobifida fusca*, a soil bacterium involved in the degradation of plant cell walls [16].

### Source Perspective Networks

In contrast to the target perspective, a source perspective network involves generating all possible functional interactions but from a particular range or collection of data sets. In this case, the goal is to constrain functional interactions so that they reflect a source-centric view for a domain of interest, such as human gut interactions. Moreover, it is possible to integrate target and source perspectives by constructing a target perspective network from a particular collection of source related data sets. This approach can be used to find functional interactions that are simultaneously target-centric and source-centric, such as cellulase interactions occurring in the human gut. In the present work a human gut microbiome [17] was used to produce two source perspective networks.

**Figure 6:**
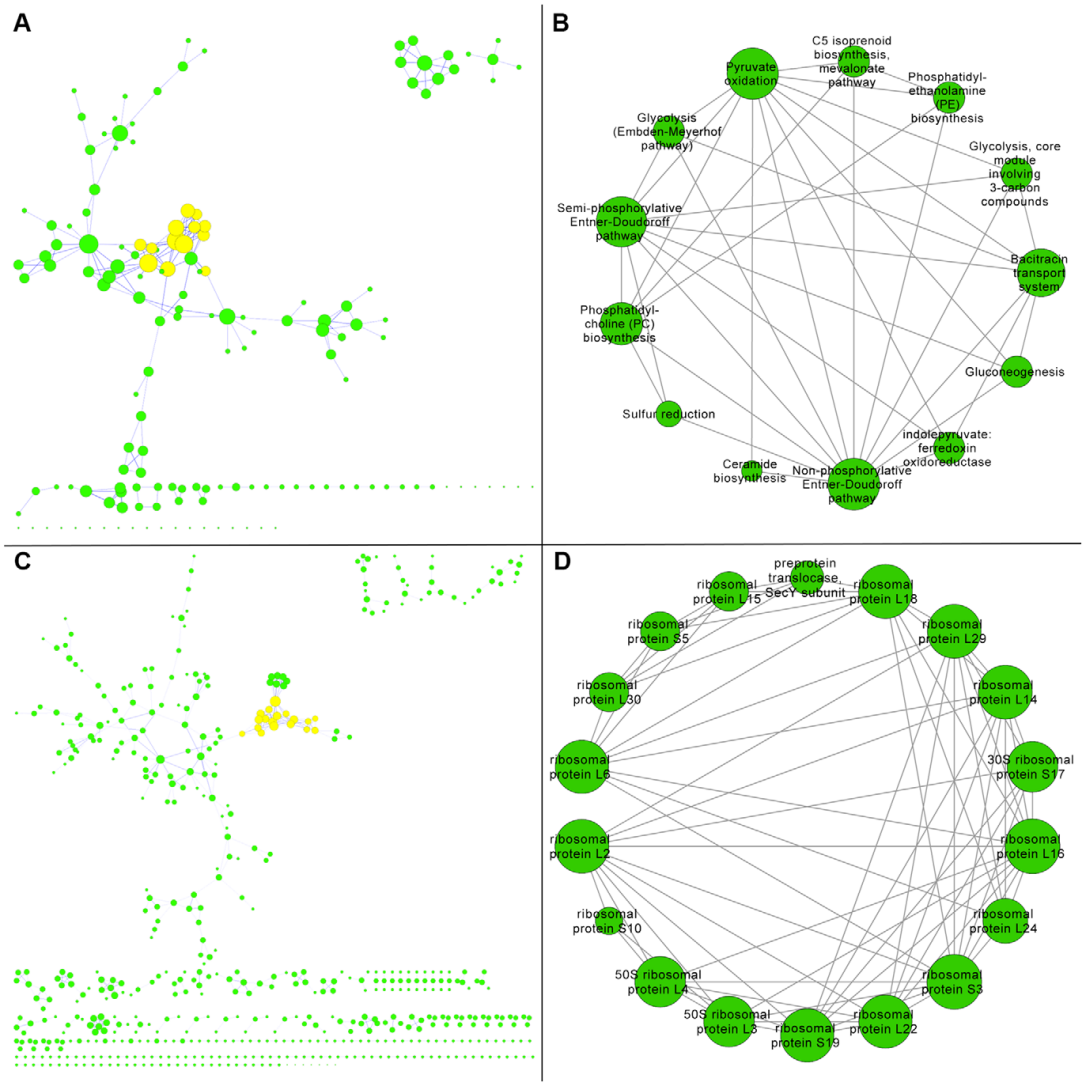
Human Gut Networks. The source perspective networks for human gut functional interactions are shown where large node diameter represents high node degree within each respective network. Panel A shows a network constructed using KEGG annotations where the highlighted nodes represent the top ranking module which is enlarged in Panel B. Panel C shows a network constructed using TIGRFAM annotations where the highlighted nodes represent the top ranking module which is enlarged in Panel D.

A KEGG gut network was constructed that consisted of 153 nodes and 192 edges (Table 1). Unlike the target perspective networks, no central hub was observed (Figure 6, Panel A). A total of 11 modules were identified within the network (Tables S1 and S2). The top ranked module (Figure 6, Panel B) scored lower than either of the top ranked cellulase modules and contained a diverse range of annotation terms (Table 2). The terms glycolysis and pyruvate occurred frequently in the pathway annotations of this module and are likely indicative of core metabolic activities across the gut community. Additional terms like isoprenoid biosynthesis and mevalonate pathway may be associated with cholesterol and possibly the statin pathway of the host liver. In fact, recent evidence suggests that the enteric microbiome can moderate response to statins [18]. Moreover, the term phosphatidylcholine biosynthesis may offer another potential link to the host liver as phosphatidylcholine from non-microbial sources has been reported to be associated with significant liver protection as part of the silybin-phosphatidylcholine complex [19].

A TIGRFAM gut network was constructed that consisted of 543 nodes and 607 edges (Table 1). Like the KEGG network, no central hub was observed (Figure 6, Panel C). A total of 57 modules were identified within the network (Tables S1 and S2). The top ranked module (Figure 6, Panel D) scored slightly higher than the top ranked KEGG module but lower than either of the top ranked cellulase modules (Table 2). The annotation term ribosomal protein dominated this module and often occurred in conjunction with the term bacterial/organelle. The result was a highly cohesive module that involved bacterial ribosomal proteins. Like the glycolysis features of the aforementioned KEGG module, this is potentially indicative of core metabolic activities across the gut community.

### Comparative Networks

Provided that two or more networks share the same perspective and a common annotative basis, it is possible to perform set theoretic operations that result in newly generated comparative networks. In the present work a second source perspective TIGRFAM network was generated using another human gut microbiome from the same study [17] that was used to produce the other source perspective networks. The TIGRFAM networks were compared to produce two new networks, an intersection network and a difference network.

**Figure 7:**
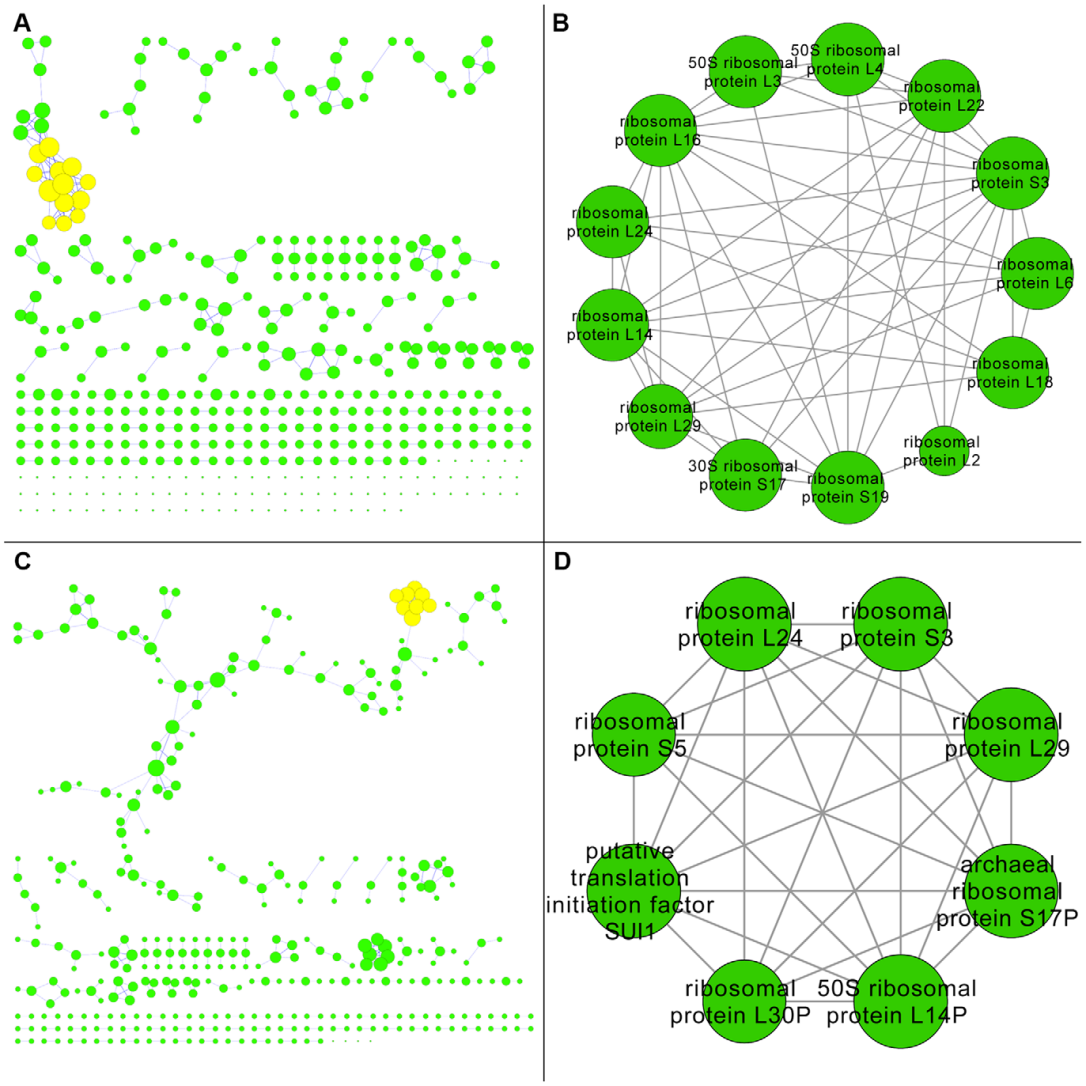
Comparative Gut Networks. The comparative networks for human gut functional interactions are shown where large node diameter represents high node degree within each respective network. Specifically, two networks were constructed using TIGRFAM annotations and compared for mutual versus exclusive nodes. Panel A shows the intersection of the networks where the highlighted nodes represent the top ranking module which is enlarged in Panel B. Panel C shows the difference of the networks where the highlighted nodes represent the top ranking module which is enlarged in Panel D.

A gut intersection network was constructed that consisted of 407 nodes and 278 edges (Table 1). This network contained a much lower ratio of edges to nodes than the non-comparative networks (Figure 7, Panel A). A total of 19 modules were identified within the network (Tables S1 and S2). The top ranked module (Figure 7, Panel B) scored the same as the top ranked TIGRFAM module (i.e. module derived using only one gut microbiome) and was composed of the same annotation terms (Table 2). In fact, the top ranked intersection module was a subset with all 13 nodes occurring in the superset of the 18 nodes that comprised the top ranked TIGRFAM module. Compared to the TIGRFAM module, the result was a reduced but highly cohesive module that similarly involved bacterial ribosomal proteins.

A gut difference network was constructed that consisted of 356 nodes and 329 edges (Table 1). This network contained a higher ratio of edges to nodes than the intersection network but was still slightly lower than the ratio in the TIGRFAM network (Figure 7, Panel C). A total of 20 modules were identified within the network (Tables S1 and S2). The top ranked module (Figure 7, Panel D) scored roughly the same as the top ranked TIGRFAM module and was composed of similar annotation terms (Table 2). While this module was also dominated by the theme of ribosomal proteins it was however more diverse and the ribosomal proteins terms frequently occurred in conjunction with the terms eukaryotic and/or archaeal, rather than bacterial.

## Discussion

The modules derived in this work are of particular interest because they represent functional metamodules. This is because it is not possible to resolve whether the activities of a single module are accomplished by a single microbial species or if they represent composite functionality produced by the greater microbial community. Therefore, metamodules provide a systems perspective at the community level. In addition, these modules provide a direct characterization of functional capability and organization as opposed to an inferred characterization on the basis of taxonomic composition. This marks an important departure from previous taxonomy driven approaches because they are susceptible to effects of community functional plasticity [20], [21] that can cloud the taxonomy versus function relationship. However, this does not exclude the incorporation of concurrent taxonomic information that could bolster the interpretation of certain data sets. As a result, metamodules can provide crucial functional insight for a variety of applied pursuits.

Modules from target perspective networks have the potential to reveal novel metabolic relationships that can subsequently assist in the hunt for new biocatalyst candidates. This process can be regarded as a metagenomic analog to the ‘guilt by association’ principle [22] that has been previously used to infer contextual information at the genomic level. In the case of the presented cellulase networks, modules that contain annotations with keywords like unknown or uncharacterized can be used to highlight genes of particular interest since annotation values (e.g. COG1699) can be easily traced back to their source genes in the raw data. This provides an expedient method to recover a shortlist of promising genes from among a raw data set that may contain tens of millions of otherwise indistinguishable records. Mining candidate genes that can be subjected to more rigorous analyses can be applied to a broad collection of interests ranging from novel glycoside hydrolase detection for biomass degradation [23] to prebiotic molecule discovery for human health applications [24].

Modules from source perspective networks have the potential to reveal how particular microbial environments orchestrate functional interactions to achieve specific functional capacities and hierarchical organization. Although gene-centric analyses have been previously applied to metagenomic functional evaluation [25], they lack the ability to provide a systems perspective of functional organization. This is because gene content analyses cannot reveal the functional interactions that are essential in understanding how various microbial communities cooperatively achieve their specific functional capabilities. In the case of the presented human gut networks, it becomes possible to speculate not only on how the gut microbiome interacts among its constituents but also on how it exerts a collective effect on host metabolic activities. Currently this is a topic of tremendous interest and many research ventures could be served by analysis and interpretation of metamodules recovered from source perspective networks. For example, metamodules from various human microbiomes could be compared to functional modules from disease related functional linkage networks [26] in order to provide complementary analyses.

The motivation for comparative networks follows logically from the utility of source perspective networks since modules from comparative networks can expose commonalities and differences in functional configurations between different data sources. Such comparisons can be used to contrast vastly different microbial environments or to find mutual cores within closely related habitats, such as the human gut of various individuals. In addition, the approach taken in the current work differs from past studies involving comparative metagenomics because it is not affected by the previously discussed limitations of taxonomy based methods and it provides information beyond the previously mentioned gene-centric analyses. In the case of the presented comparative gut networks, it is possible to see that essential core modules could be developed for a variety of human microbiomes by deriving respective intersection networks from sets of multiple participants. The ability to directly contrast and compare metagenomic functional repertoires can offer tremendous utility to existing comparative research areas such as obese versus lean gut microbiomes [27] and control versus autistic gut microbiomes [28].

The implementation presented here was based on several simplifications and assumptions that could be addressed by future works. The use of transitive functional interactions favoured the formation of complete subgraphs (i.e. a component where each node has an edge to every other node). Although this was done to maximize functional information, it could also have contributed to an inflation of network edges that can bias module-finding algorithms. Other implementations should consider the prospect of constrained transitivity as a comparison. Similarly, the confidence thresholds for defining operons should be further tested in a metagenomic context and this could be performed in conjunction with limits for transitivity in order to characterize the interaction of these two essential factors. Further still, the operon reference data obtained from RegulonDB [29] represents knowledge derived from a classic model organism. Given that metagenomes offer access to the uncultured microbial majority, the applicability of such reference data remains to be established. In general, an improved understanding of the properties of metagenomic operons (e.g. size, composition, frequency, etc.) would benefit metagenomic annotation networks and related interests.

## Methods

Metagenomic genes were parsed from downloaded raw data and used in a two-phase protocol consisting of network prediction followed by network translation. All operations were computationally implemented in Java and run on a Gateway NV59 laptop using an Intel Core i3-330 M processor.

### Data Preparation

The raw data consisted of the complete set of public metagenomes available from the Integrated Microbial Genomes with Microbiome samples (IMG/M) metagenomics database [13] as of late August 2011. This included 224 data sets comprised of 40,189,394 total genes, distributed across 40,325,419 scaffolds. The simulated data sets (simLC, simMC, simHC) were removed, as well as any datasets that did not contain gene coordinate information (DRU, VLU, Yorkshire Pig Fecal Sample 266, Yorkshire Pig Fecal Sample 267), since these coordinates are required for the network prediction phase (see Network Prediction). The remaining 217 datasets included 39,660,386 total genes, from which 207,097 rRNA genes were excluded, leaving an aggregate working data set of 39,453,289 protein-coding genes.

We selected the IMG/M as data source for three reasons: (i) it offered a very large amount of data from a diverse range of environments (Figure 1); (ii) virtually all of the annotated genes (>99.5%) included information about their position and strand within the scaffolds in which they occurred; (iii) there was a high proportion of sufficiently assembled scaffolds such that multiple genes could occur within a single scaffold. This is in stark contrast to repositories that primarily offer data from short reads that frequently lack a single gene, let alone multiple genes. Overall, these factors are indicative of a current dichotomy in sequence databases: submitter-biased, such as MG-RAST [30], which cater to needs of authors that require a public depository of their data; *versus* query-biased, such as the IMG/M, which are focused on offering the expedient retrieval of data.

### Network Prediction

Operons were predicted in scaffolds containing two or more adjacent genes in the same strand (Figure 2, Panel A) using a previously published method based on intergenic distances [*D*  =  *gene2_start* − (*gene1_end* +1)], where the likelihood for two genes to be in the same operon given the distance between them is assigned based on the ratio of known genes in operons to known genes in different transcription units found at such distance [31], [32], [33]. A minimum threshold of confidence was selected that is equivalent to a positive predictive value of 0.85 (meaning that 85% of the predictions are expected to consist of true positives), as evaluated against known operons of *Escherichia coli* K12 found in RegulonDB [29]. Next, functional interactions were defined in a pairwise manner for all members of a predicted operon. For example, an operon with the consecutive gene members *a*, *b*, and *c* would yield predicted functional interactions for the adjacent pairs *ab* and *bc*, plus an additional transitive functional interaction, namely *ac*. In the case of target perspective networks (see Results) operons were filtered according to the presence or absence of a target annotation by requiring a minimum number of member genes to contain a specific keyword descriptor (Figure 2, Panel B). The effects of target stringency (i.e. the size of the minimum number) were also tested (see Results).

### Network Translation

Each gene in a given operon was mined for the following types of functional annotations: MetaCyc pathways [34], COGs [35], KEGG pathways [36], and TIGRFAMs [37]. A gene may have multiple annotation types and also have multiple values for a given type (Figure 2, Panel C). For each operon, the obtained functional annotations were used to infer functional interactions for annotations having the same type but different values. Translated interactions were inferred directly for immediately adjacent gene pairs but also transitively for downstream members within the same operon (Figure 2, Panel D). Note that the use of transitive translations is necessarily a reflection of the transitivity implemented in the network prediction phase.

The interactions were sorted by annotation type in order to derive a collection of discrete annotation networks for any given data source where each network had a particular annotative basis, such as MetaCyc or COG. This was possible because the translation of interacting genes into interacting annotations generated a unique set of nodes and edges with respect to each of the annotative bases. Moreover, specific annotation values (e.g. COG1363) were considered to be synonymous with their textual descriptors (e.g. cellulase M and related proteins) thereby providing a means for the conversion of nodes into a more verbose form. We note that it would have been possible to use the descriptors that were already available in the source data, rather than using the categorized annotations. The raw descriptors were not used in order to contrast the differences between specific annotative bases and also to avoid inflation caused by the redundant duplication of synonymous descriptors that varied only in terms of minor formatting features (i.e. lexicographical redundancy). Moreover, using specific categorized annotations produced connections between otherwise disjoint subgraphs thereby yielding a more connected network. However, future works may utilize the raw descriptors if the goal is to create a single global network of annotation linkages, regardless of annotation category.

### Conclusion

Metagenomic annotation networks offer a novel taxonomy-free approach for understanding the functional capacity and hierarchical organization of integrated microbial communities. In particular, these networks can be analyzed for functional metamodules that subsequently provide a systems perspective at the microbial community level. Modules from target perspective networks can be used to infer interactions for a given gene or protein of interest. In turn, these interactions can be instrumental in revealing novel metabolic relationships that can subsequently assist in the hunt for new biocatalyst candidates. Modules from source perspective networks reveal how particular microbial environments orchestrate functional interactions to achieve specific functional capacities and hierarchical organization. This offers a mechanism of functional characterization that goes beyond gene-centric analyses. Modules from comparative networks can expose commonalities and differences between functional configurations from different data sources. These comparisons can be used to contrast vastly different microbial environments or to find mutual cores within closely related habitats, such as the human gut of various individuals. Comparing the functional repertoire of human microbiomes will be especially informative for future works of medical interest.

## Supporting Information

Table S1 Functional keys of genes in clusters analyzed in this work.
(XLS)

Table S2 Functional annotations for clusters analyzed in this work.
(XLS)
